# Pigmented microcystic chromophobe renal cell carcinoma in a patient with end-stage kidney disease: Importance of evaluating non-neoplastic renal parenchyma

**DOI:** 10.1016/j.eucr.2026.103529

**Published:** 2026-07-01

**Authors:** Adam Nedoroscik, Jan Gajdos, Vladimir Borovicka, Martin Kment, Marek Kollar

**Affiliations:** a Institute of Pathology General University Hospital in Prague, First Faculty of Medicine, Prague, Czech Republic; b Department of Pathology, University of Pavol Jozef Šafárik in Košice, Medical Faculty, Košice, Slovak Republic; c Transplantation Surgery Department, Institute of Clinical and Experimental Medicine, Prague, Czech Republic; d The Clinical and Transplant Pathology Centre, Institute of Clinical and Experimental Medicine, Prague, Czech Republic

**Keywords:** Chromophobe renal cell carcinoma, Microcystic variant, Focal segmental glomerulosclerosis, Nephrectomy, Renal pathology

## Abstract

Adenomatous pigmented microcystic chromophobe renal cell carcinoma (PMCRCC) is a rare variant of chromophobe renal cell carcinoma. We report a 72-year-old male with end-stage chronic kidney disease undergoing transplant evaluation, in whom PMCRCC and concomitant focal segmental glomerulosclerosis (FSGS) were identified after nephrectomy. The tumor showed characteristic microcystic architecture, focal pigmentation, and immunophenotypic features. To our knowledge, this is among the few reported cases of PMCRCC with detailed evaluation of the accompanying non-neoplastic renal parenchyma. The case highlights the characteristic features of this rare tumor variant and illustrates the value of systematic assessment of non-neoplastic kidney tissue in nephrectomy specimens

## Highlights

• Rare pigmented microcystic chromophobe renal cell carcinoma with concurrent focal segmental glomerulosclerosis.
• Thorough assessment of non-neoplastic renal tissue can reveal clinically significant coexisting kidney disease.
• A multidisciplinary nephropathology approach supports personalized management and transplant evaluation.

## 1 Introduction

Chromophobe renal cell carcinoma (CRCC) accounts for approximately 5–7% of renal epithelial malignancies and is characterized by distinctive morphological and immunohistochemical features. A rare variant, adenomatous pigmented microcystic chromophobe renal cell carcinoma (PMCRCC), was first described in 1998 and remains poorly documented in the literature.^1^

PMCRCC is characterized by microcystic and adenomatous architectural patterns, pigmentation, oncocytic cytoplasm, prominent cell membranes, and characteristic nuclear features. Immunohistochemically, these tumors typically express epithelial markers including EMA, cytokeratins, and CD117, with focal CK7 positivity. PMCRCC seems to have a better prognosis compared to other renal cell carcinomas. Distant metastases and sarcomatoid transformation are usually absent 2, 3, 4.

Focal segmental glomerulosclerosis (FSGS) is one of the most common causes of nephrotic syndrome in adults and may also present with non-nephrotic proteinuria. Immunofluorescence (IF) is typically negative, with occasional nonspecific segmental IgM and/or C3 trapping in sclerotic glomeruli 5, 6, 7. FSGS represents a pattern of glomerular injury that may be primary or secondary to various conditions. Differentiation between these forms remains challenging and relies on integration of clinical and pathological findings rather than on a single definitive criterion. The remaining parts of the glomerular tuft in the same glomerulus are virtually intact by light microscopy. Columbia classification categorizes morphologic variants of FSGS,^7^ although it is not specific for pathogenesis. In addition, immunohistochemical markers have been described as potential predictors of disease progression.^8^

We present a case of PMCRCC arising in a patient with advanced chronic kidney disease and secondary FSGS, emphasizing both the diagnostic features of this rare tumor variant and the importance of evaluating non-neoplastic renal parenchyma in nephrectomy specimens.

## 2 Case presentation

### 2.1 Clinical data

A 72-year-old Caucasian male with end-stage chronic kidney disease (CKD stage 5) of unknown origin, undergoing assessment for kidney transplantation, was incidentally found to have a renal mass on computed tomography performed as part of routine pre-transplant evaluation (Fig. 1). His medical history included hypertension, hyperlipidaemia, peripheral arterial disease, and benign prostatic hyperplasia. Laboratory tests revealed markedly elevated serum creatinine (654 μmol/L). The patient underwent laparoscopic left nephrectomy.

**Fig. 1:**
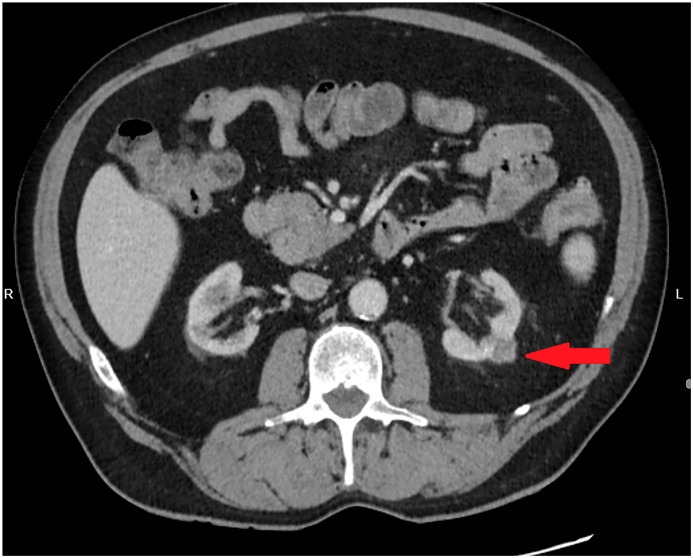
Contrast-enhanced computed tomography (CT) of the abdomen. Axial contrast-enhanced CT image demonstrating a small subcapsular lesion in the left kidney (arrow).

### 2.2 Pathological examination

#### 2.2.1 Macroscopic findings

A 12 × 9 × 8 mm subcapsular multicystic tumor with a brown cut surface and focal black pigmentation was identified. The surrounding nonneoplastic renal parenchyma was markedly thinned to 5 mm.

#### 2.2.2 Microscopy of the tumor

Microscopically, the tumor showed a predominantly microcystic and microalveolar architecture with focal adenomatous arrangements. Tumor cells had oncocytic cytoplasm, distinct cell membranes, and characteristic „raisinoid“ nuclei with perinuclear halos. Brown pigment was present both intracellularly and extracellularly. No necrosis, vascular invasion, or extension into perirenal fat was present (Fig. 2).

**Fig. 2:**
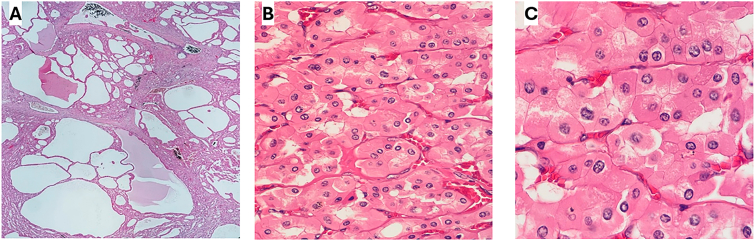
Hematoxylin and eosin (H&E) staining of the tumor. (A) Predominantly microcystic and adenomatous architecture (magnification 40x). (B) Oncocytic tumor cells with focal “raisinoid” nuclei (magnification 200x). (C) Detail of tumor cells with abundant oncocytic cytoplasm and prominent cell membranes (magnification 400x).

Immunohistochemically, tumor cells were diffusely positive for PAX8, CK AE1/AE3, CD117, EMA, and CAM5.2, with focal CK7 positivity. On the other hand, RCC marker, CD10, vimentin, HMB-45, and Melan A were completely negative (Fig. 3). Special stains (Masson–Fontana and Schmorl) confirmed the presence of melanin pigment (Fig. 4).

**Fig. 3:**
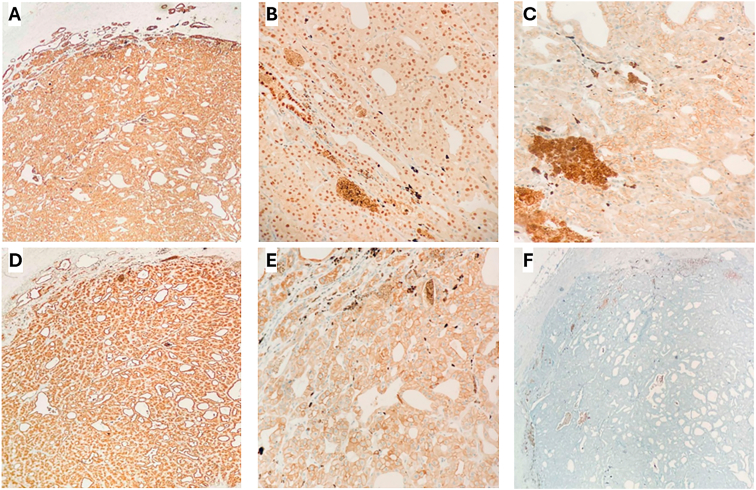
Immunohistochemical profile of the tumor. (A) Positivity for cytokeratin AE1/AE3 (magnification 40x). (B) Positivity for PAX8 (magnification 100x). (C) Positivity for CD117 (c-KIT) (magnification 100x). (D) Positivity for epithelial membrane antigen (EMA) (magnification 40x). (E) Positivity for CAM5.2 (magnification 40x). (F) Negativity for HMB-45 (magnification 40x).

**Fig. 4:**
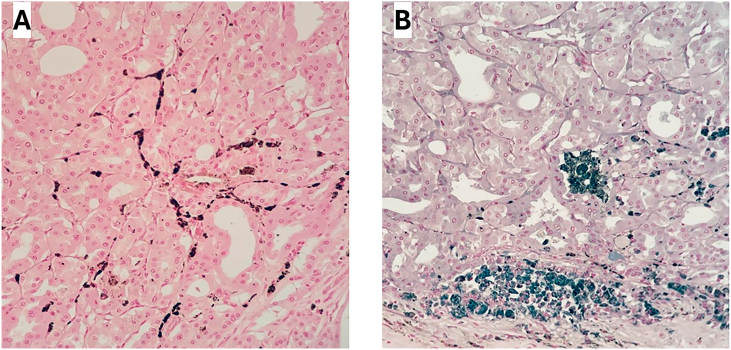
Special histochemical staining of tumor pigment. (A) Positive Masson–Fontana stain in pigment (magnification 100x). (B) Positive Schmorl stain in pigment (magnification 100x).

#### 2.2.3 Microscopy of the non-neoplastic renal parenchyma

The non-neoplastic kidney demonstrated significant vascular changes, including fibrointimal thickening and arteriolar hyalinosis. Approximately 50% of glomeruli were globally sclerotic (often in subscapular region), while others showed segmental sclerosis without specific anatomic predilection. Interstitial fibrosis involved approximately 50% of the parenchyma, accompanied by chronic inflammation (Fig. 5).

**Fig. 5:**
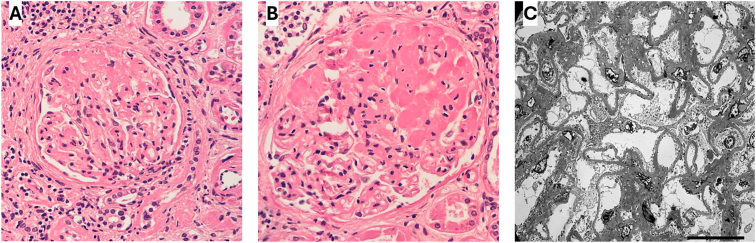
Non-neoplastic renal parenchyma (H&E staining). (A) Glomerulus with segmental sclerosis - H&E staining (magnification 200x). (B) Glomerulus with segmental sclerosis - H&E staining (magnification 400x). (C) Transmission electron microscopy of non-neoplastic renal parenchyma demonstrating partial podocyte foot process effacement without electron-dense deposits.

Immunofluorescence was negative for IgG, IgA, IgM, C3, C1q, kappa, lambda, and amyloid A. Immunohistochemical analysis showed C4d and CD44 strong positivity in glomeruli and also in areas with segmental sclerosis. Electron microscopy revealed partial podocyte foot process effacement without immune deposits (Fig. 5).

These findings were consistent with secondary FSGS (NOS variant), most likely related to vascular pathology.

### 2.3 Follow-up

Postoperative imaging showed no evidence of recurrence or metastasis. The patient was re-listed for kidney transplantation. At this time, it is already known that chronic renal insufficiency was caused by focal segmental glomerulosclerosis (FSGS), most likely a secondary form due to vascular etiology.

## 3 Discussion

We report a case of PMCRCC in a 72-year-old male patient with end-stage chronic kidney disease, concomitant FSGS in non-neoplastic parenchyma, in whom the tumor was incidentally detected during pre-transplant evaluation without a previously established underlying casuse of renal failure. The diagnosis was established based on classic morphological and immunohistochemical findings. In addition, detailed evaluation of non-neoplastic renal parenchyma revealed secondary FSGS associated with significant vascular changes.

PMCRCC is a rare variant of CRCC with distinctive morphological features that may pose diagnostic challenges. Its microcystic and adenomatous architecture, combined with pigmentation, may lead to confusion with other oncocytic renal neoplasms.^2^

Other studies found in some tumors bigger multicystic spaces, which did not usually form majority of tumor mass. In four out of 32 tumors, they found regions of typical CRCC with solid-alveolar patterns.^9^ In addition, the typical tumor component described in literature and also in our case is pigmentation, macroscopically evident as multiple small gray to black dots.^4^^,^^9^^,^^10^ Our immunohistochemical findings are consistent with those previously reported. Diffuse expression of PAX8, CD117, CK AE1/AE3 and CAM5.2 supports the diagnosis of chromophobe renal carcinoma. On the other hand, CK7 was focally positive which helps us to distinguish it from classic chromophobe RCC, oncocytoma, and other oncocytic tumors, while chromophobe RCC is usually CK7 positive.

Although the presence of secondary FSGS is not unexpected in a patient with advanced chronic kidney disease, its recognition remains clinically relevant. Previous studies have shown that non-neoplastic renal diseases are frequently underreported or overlooked in tumor nephrectomy specimens. Detailed assessment of the non-neoplastic renal parenchyma may provide important information regarding the etiology of renal dysfunction and may contribute to clinical management.^11^

In this patient, the presence of secondary FSGS likely contributed to chronic kidney disease and has implications for post-transplant management. The vascular changes observed suggest a secondary form of FSGS, supported by the absence of immune deposits and only partial podocyte foot process effacement. In addition, immunohistochemical analysis demonstrated expression of CD44 and C4d in both normal and sclerotic glomerular areas. Zagorec N et al. (2024) showed that CD44 and C4d deposition represent an independent clinical-pathological predictor of disease progression in patients with FSGS.^12^ CD44 is not specific for distinguishing primary from secondary FSGS, as its expression may be observed in both forms; however, it has been associated with worse prognosis.^13^ Moreover, CD44 expression is typically present in FSGS, whereas it is absent in minimal change disease, which may aid in the differential diagnosis in challenging cases.^14^

## 4 Conclusion

This report describes the characteristic morphologic and immunophenotypic features of PMCRCC, a rare variant of chromophobe renal cell carcinoma. In addition, the case illustrates the importance of careful evaluation of non-neoplastic renal parenchyma, which may reveal clinically relevant renal pathology even in nephrectomy specimens removed for tumor assessment.

## Ethical approval

This study did not require approval by an ethics committee, as it involved only anonymized data and did not include identifiable patient information.

## Sources of funding

This research did not receive any specific grant from funding agencies in the public, commercial, or not-for-profit sectors.

## CRediT authorship contribution statement

**Adam Nedoroscik:** Conceptualization, Visualization, Writing – original draft. **Jan Gajdos:** Formal analysis, Resources, Supervision. **Vladimir Borovicka:** Data curation, Formal analysis, Supervision. **Martin Kment:** Conceptualization, Resources, Writing – review & editing. **Marek Kollar:** Data curation, Formal analysis, Funding acquisition, Writing – review & editing.

## Conflict of interests

Except for employment with the healthcare provider that provided the healthcare, we do not declare any other conflict of interest.
